# JcLEA, a Novel LEA-Like Protein from *Jatropha curcas*, Confers a High Level of Tolerance to Dehydration and Salinity in *Arabidopsis thaliana*

**DOI:** 10.1371/journal.pone.0083056

**Published:** 2013-12-31

**Authors:** Jing Liang, Mingqi Zhou, Xin Zhou, Yuanjie Jin, Ming Xu, Juan Lin

**Affiliations:** State Key Laboratory of Genetic Engineering, Institute of Plant Biology, School of Life Sciences, Fudan University, Shanghai, People's Republic of China; Purdue University, United States of America

## Abstract

*Jatropha curcas* L. is a highly drought and salt tolerant plant species that is typically used as a traditional folk medicine and biofuel crop in many countries. Understanding the molecular mechanisms that underlie the response to various abiotic environmental stimuli, especially to drought and salt stresses, in *J. curcas* could be important to crop improvement efforts. In this study, we cloned and characterized the gene for a late embryogenesis abundant (LEA) protein from *J. curcas* that we designated JcLEA. Sequence analyses showed that the JcLEA protein belongs to group 5, a subgroup of the LEA protein family. In young seedlings, expression of *JcLEA* is significantly induced by abscisic acid (ABA), dehydration, and salt stress. Subcellular localization analysis shows that that JcLEA protein is distributed in both the nucleus and cytoplasm. Moreover, based on growth status and physiological indices, the overexpression of *JcLEA* in transgenic *Arabidopsis* plants conferred increased resistance to both drought and salt stresses compared to the WT. Our data suggests that the group 5 JcLEA protein contributes to drought and salt stress tolerance in plants. Thus, *JcLEA* is a potential candidate gene for plant genetic modification.

## Introduction

Water and ion concentrations in soil are important abiotic elements for living organisms. Unlike animals, plants are sessile organisms that are exposed to seasonal and local environmental variations, and their survival and growth are strongly influenced by local stress factors. It has been estimated that 70% of crop yield losses are caused by abiotic stresses, with drought and high salinity being the most serious threats to crop production in many areas of the world [1]. Plants have developed multi-pathway survival strategies, at multiple-levels and on multiple-scales to allow them to grow and reproduce successfully. The late embryogenesis abundant (LEA) proteins are involved in one type of self-protection mechanism. LEA proteins belong to a large protein family that is closely associated with resistances to abiotic stresses, especially to drought, in a range of organisms [2]. The LEA proteins were originally identified in terrestrial plants, including wheat (*Triticum aestivum*) and cotton (*Gossypium hirsutum*), and were subsequently detected in many other species of higher plants [3]–[8]. LEA proteins mainly accumulate in the late stages of embryogenesis in plant seeds under dehydration stress [5], and have also been found in bacteria [9], slime molds [10], nematodes [11]–[15], fungi [16], and humans [17].

LEA proteins are localized to various subcellular compartments, including the nucleus, mitochondria, chloroplast, endoplasmic reticulum, vacuole peroxisome, plasma membrane and cytoplasm [18], [19]. They function as a protecting factor associated with the dessication tolerance of seeds, pollen and anhydrobiotic tissues [20], [21]. Over the past two decades, remarkable research progress has been achieved in the characterization of LEA proteins, which are considered to be functional effectors in the generation of abiotic tolerance in plants.

The LEA protein super families have a diversity of sequences and functions. Based on the nomenclature introduced by Battaglia, LEA proteins are categorized into seven distinctive groups, which include groups 1 (LEA-5), 2 (dehydrin), 3 (LEA-4), 4 (LEA-1), 5 (A: SMP; B: LEA-3; C: LEA-2), 6 (LEA-6), and 7 (ABA-WDS) [22]. Groups 1, 2, 3, and 4, which share specific sequence motifs within each group, are considered to be typical or genuine hydrophilic LEA proteins, while group 5 LEAs lack significant signature motifs or consensus sequences and are regarded as atypical LEA proteins, containing a significantly higher proportion of hydrophobic residues than typical LEA proteins [22]. It is noteworthy that group 5 proteins are natively folded and are not soluble after boiling, suggesting that they are probably not heat stable [23]. The average molecular weight of the group 5 proteins is 18.1 kDa, and most group 5 LEA proteins are acidic. Based on these features, it is predicted that group 5 proteins play roles in seed maturation, dehydration, and the combining of concentrated ions [8]. At present, a number of group 5 *LEA* genes have been cloned and characterized from many plant species, including cotton LEA D-34 [24], maize RAB28 [25], carrot ECP31 [26], Arabidopsis AtECP31 and AtRAB28 [27], [28], *Craterostigma plantagineum* pcC27-45 [29], tomato ER5 [30], cayenne pepper CaLEA6 [31], *Medicago truncatula* MtPM25 [32], soybean GmPM22, GmPM24, GmPM25, and GmPM26 [33], and OsLEA5 from rice [34]. Nevertheless, group 5 still attracts fewer investigations compared with the other LEA subcategories mentioned above.

*Jatropha curcas* L. is a multi-purpose shrub belonging to the plant family *Euphorbiaceae*. The species is widespread throughout arid and semi-arid tropical regions of the world, and has been utilized as a source of traditional folk medicines. *J. curcas* is so highly tolerant to dehydration that it can survive 30% of the field capacity (FC) without drought injury [35]. It has been reported that *J. curcas* has an efficient adaptive mechanism that enables it to tolerate severe drought situations by maintaining leaf water status and making effective osmotic adjustments [36]. Some evidence also suggests that *J. curcas* possesses a moderate tolerance to salinity, because the seedlings can tolerate up to 30 mM NaCl (in irrigation water) without negative effects on their growth parameters. Salt-treated plants (irrigated with NaCl levels of over 30 mM NaCl) showed a significant reduction in growth (by 5.82% for every 10 mM increment in NaCl concentration). This finding shows that *J. curcas* was more saline-tolerant than most of the typical Mediterranean crops [37], [38]. However, the molecular mechanisms involved in the response of *J. curcas* to environmental stresses, especially the functions of LEA proteins in protecting seedlings from the effects of dehydration and high salinity, are still poorly understood. In the present report, we document the molecular cloning of an *LEA*-like gene from *J. curcas* by RACE-PCR. Bioinformatics and expression analyses reveal that the *J. curcas* LEA protein strongly resembles group 5 LEAs from other plant species. The results of physiological assays under drought and salt treatments revealed that the *JcLEA* gene confers significantly enhanced tolerance to drought and salt in transgenic *Arabidopsis* plants. Based on these results, the potential application of the *JcLEA* gene in genetic engineering of crops is discussed.

## Materials and Methods

### Plant Materials and Sampling Locations

Seeds of *Jatropha curcas* were obtained from the *Jatropha curcas* Germplasm Resources Preservation Center. The plants were grown in a natural environment on farmland in the Renhe (Panzhihua, Sichuan Province, China). No specific permission was required for any of the locations or activities, and the field studies did not involve endangered or protected species. Seeds were germinated in soil in the greenhouse. Roots, leaves and stems were collected and stored at −70°C. Seeds of *Arabidopsis thaliana* accession Columbia (Col-0) were obtained from the *Arabidopsis* Biological Resource Center (ABRI: Columbus, OH, USA).

### Plant Growth Conditions

For gene expression analysis, 30-day-old *J. curcas* plants were treated with 300 mM NaCl for 1 day, 100 µM ABA (Abscisic acid) for 3 hours and 30% PEG-4000 for 3 days, respectively. For root length determination, surface-sterilized *Arabidopsis* seeds were germinated on MS (Murashige and Skoog) medium (pH 5.8) containing 2% phytagel with 50 mM, 100 mM, and 150 mM NaCl or 10% and 15% PEG-4000. The seedlings were grown under a 16 h light/8 h dark photoperiod at a temperature of 22°C with a light intensity of 150 µmol m^−2^ s^−1^ in a plant growth cabinet. Plants were exposed to drought and salt stress for 14 days and then photographed, and root length was measured using ImageJ software.

Two transgenic *Arabidopsis* lines overexpressing *JcLEA* were used for physiological tests of tolerance to high salinity and dehydration. Seedlings growing in the greenhouse under a 16 h light/8 h dark cycle at 22°C for 4 weeks were watered with 50 mM, 100 mM, 150 mM, or 200 mM NaCl for 25 days and photographed and analyzed for survival rates at the 25^th^ day of salt treatment. For drought treatment, water was withheld from 4-week-old plants for 14 days, after which they were rewatered for 10 days. The survival rate determinations and physiological measurements were carried out at the 14^th^ day under the drought treatment and the 10^th^ day after rewatering. Physiological measurements including the relative water content, electrolyte leakage, glucose content, sodium content, and potassium content were measured at the 14^th^ day of the dehydration treatment, the 10^th^ day after rewatering, and the 25^th^ day of salt treatment. Whole plants were used for investigation of sodium content and potassium content, and leaves were used for the other physiological measurements.

### RNA Extraction

Total RNA of *J. curcas* was extracted as previously reported [39]. Total RNA was extracted from *A. thaliana* leaves using the Plant RNA Mini Kit (Aidlab Biotechnologies Co., Inc., Ltd, China). RNA quality and concentration were analyzed by agarose gel electrophoresis (EC250-90, E-C Apparatus Corporation) and spectrophotometry (WFZUV-2100, Unico™ Instruments Inc.). The RNA samples were stored at −70°C prior to use.

### Cloning of *JcLEA* Gene from *J. curcas*

Primers LEAF and LEAR were used in a reverse transcription polymerase chain reaction (RT-PCR) to obtain a partial fragment of the *JcLEA* cDNA. This pair of primers corresponded was designed from highly conserved amino acid sequences of LEA proteins from *Ricinus communis* (XP_002520716), *Glycine max* (XP_003536910), *Medicago truncatula* (XP_003590628) and *Gossypium hirsutum* (P09444). Amplifications were performed at 94°C for 4 min, followed by 30 cycles of amplification (94°C for 40 s, 55°C for 30 s, and 72°C for 40 s). Following this, 5′- and 3′-rapid amplifications of cDNA ends were conducted using SMART technology (SMART™ RACE cDNA Amplification Kit) to produce a full-length *JcLEA* cDNA. The *JcLEA* cDNA was sequenced using the DYEnamic Direct dGTP Sequencing Kit (Amersham Pharmacia, UK) and a 373A DNA sequencing instrument. The sequences were then compared with known sequences in the NCBI database using blastp (Standard Protein-Protein BLAST) on NCBI (www.ncbi.nlm.nih.gov). The conserved domains were searched with RPS-BLAST (Search the Conserved Domain Database) in the NCBI database. The phylogenetic tree of LEA proteins sequences was constructed based on their conserved properties and homology. The representative protein sequences of seven previously reported groups of LEA proteins were obtained from the NCBI database (http://www.ncbi.nlm.nih.gov/). MEGA 5.0 was used as a computational phylogenetics tool to construct the maximum likelihood tree [40]. The classification of LEA groups was performed as previously described [22]. The sequences of all primers used in this paper are listed in Table 1.

**Table 1:** Oligonucleotide primers and their uses in this study.

Primers	Sequence	Usage
LEAF	5′-ATHACNATHGGWGARGCWTTRGAR-3′ (H is A, C and T; N is A, C, G and T; W is A and T; R is A and G)	Cloning of *JcLEA* cDNA
LEAR	5′-RSWAGCAGCAACDCCDCCWGG-3′ (W is A and T; D is A, G and T)	Cloning of *JcLEA* cDNA
JcLEAF1	5′-GACTACCCTCTCTGATGTTTTAGCGGAC-3′	Cloning of *JcLEA* cDNA
JcLEAF2	5′-GATGCTGAAGGTGTGATTGGAGCGGAAA-3′	Cloning of *JcLEA* cDNA
JcLEAR1	5′-CAGCATCATCCCGAGTTACCGTCTTGTC-3′	Cloning of *JcLEA* cDNA
JcLEAR2	5′-CAGCCACAGAAGCAGCAACTCCACCAGG-3′	Cloning of *JcLEA* cDNA
JcLEAfull-F	5′-ATGAGCCAGGGGCAACCACGAAGAA-3′	Cloning of *JcLEA* cDNA, Semi-quantitative PCR, Transformants identification
JcLEAfull-R	5′-TTATGGGTTCTGATTAAGCCTAGCAGCTGC-3′	Cloning of *JcLEA* cDNA, Semi-quantitative PCR, Transformants identification
JcLEAQ1	5′-ACGCGGGAACAGAGGTGGCCACTGC-3′	Cloning of *JcLEA* cDNA
JcLEA-B	5′-GCggatccATGAGCCAGGGGCAACCAC-3′	Cloning of *JcLEA* cDNA
JcLEA-X	5′-GCtctagaTTATGGGTTCTGATTAAGC-3′	Cloning of *JcLEA* cDNA
LEA-GFPF	5′-AAccatggTAATGAGCCAGGGGCAACCA-3′	Construction of JcLEA-GFP
LEA-GFPR	5′-GGactagtTGGGTTCTGATTAAGCCTAGCA-3′	Construction of JcLEA-GFP
3′-RACE CDS Primer	5′-AAGCAGTGGTATCAACGCAGAGTAC(T)30N-1N-3′	Cloning
5′-RACE CDS Primer	5′-(T)_25_N_-1_N-3′	Cloning of *JcLEA* cDNA
UPM	Long: 5′-CTAATACGACTCACTATAGGGCAAGCAGTGGTATCAACGCAGAGT-3′ Short: 5′-CTAATACGACTCACTATAGGGC-3′	Cloning of *JcLEA* cDNA
NUP	5′-AAGCAGTGGTATCAACGCAGAGT-3′	Cloning of *JcLEA* cDNA
SMARTIIA Oligo	5′-AAGCAGTGGTATCAACGCAGAGTA-3′	Cloning of *JcLEA* cDNA
AP	5′-GGCCACGCGTCGACTAGTACTTTTTTTTTTTTTTTTT-3′	Cloning of *JcLEA* cDNA
JcActin-rF	5′-TAATGGTCCCTCTGGATGTG-3′	Quantitative Real-time PCR
JcActin-rR	5′-AGAAAAGAAAAGAAAAAAGCAGC-3′	Quantitative Real-time PCR
LEA-R1	5′-AGTGATGTTGTTAGAGAA-3′	Quantitative Real-time PCR
LEA-R2	5′-CCATTGTAATATCCATACC-3′	Quantitative Real-time PCR

### Plant Transformation Constructs

The open reading frame (ORF) fragment of *JcLEA* was amplified using the gene-specific primers JcLEA-B and JcLEA-X and Pfu DNA polymerase (Promega). The resulting fragment was digested with *Bam*HI/*Xba*I and ligated into the corresponding restriction sites of the pCAMBIA1304 vector under control of the *CaMV*35S promoter. For production of the JcLEA-GFP fusion protein, the *JcLEA* coding region was amplified using primers LEA-GFPF and LEA-GFPR. The PCR product was digested with *Nco*I/*Spe*I and inserted into the pCAMBIA1302 vector. Expression of the JcLEA-GFP protein fusion was driven by the *CaMV35S* promoter. These constructs were sequenced to confirm the amplification fidelity and the exact insertion point of *JcLEA* in the pCAMBIA vectors.

### Plant Transformation and Confirmation

For stable transformation of *Arabidopsis*, *Arabidopsis* Col-0 was transformed using the Agrobacterium-mediated floral dip method. *Agrobacterium tumefaciens* strain LBA4404 carrying the plasmid *35S::JcLEA* was cultured overnight to an OD_600_ of 1.2 to 1.6. The *Agrobacterium* cells were collected by centrifugation for 10 min at room temperature at 5000 rpm and then resuspended to an OD_600_ of approximately 0.8 in floral dip inoculation medium (2.16 g l^−1^ MS powder, 0.5 g l^−1^ 2-(N-Morpholino) ethanesulfonic acid, 50 g l^−1^ sucrose, 10 µg l^−1^ 6- benzylaminopurine and 0.02% v/v silwet L-77, pH 5.7). The T_1_ plants were selected on hygromycin (30 mg l^−1^) and further confirmed by PCR using primers JcLEA-full-F and JcLEA-full-R. T_2_ lines showing 3∶1 segregation were carried forward to the T_3_ generation. PCR positive homozygous T_3_ and T_4_ lines were used for functional analyses of *JcLEA*.

For transient gene expression in *Nicotiana tabacum* cells, leaves of 4-week-old sterilized tobacco plants were infiltrated with *A. tumefaciens* GV3101 carrying the JcLEA-GFP fusion vector. The leaves were placed on MS solid medium in the dark at 25°C for two days before the imaging analysis.

### Fluorescence and Luminescence Imaging

Fluorescence signals were imaged using a confocal laser scanning microscope (Zeiss 710, Germany) at a magnification of 20× and analyzed with Zen software. Before the detection of the fluorescence signals, the tobacco leaf pieces were stained with DAPI for 40 min to facilitate the observation of leaf cell nuclei.

### Western Blot Analysis

Plant nuclear and cytosolic proteins were extracted from *N. tabacum* leaf disks using a Nuclear and Cytoplasmic Protein Extraction Kit (Shanghai Sangon Biotech, China). A 50 ng sample of the extracted protein was boiled in SDS gel loading buffer and electrophoresed on a 15% polyacrylamide gel for three hours. Proteins were then electroblotted onto PVDF membrane using the Mini Trans-Blot® Electrophoretic Transfer Cell System (Bio-Rad). The membranes were then blocked in Tris-HCl-buffered saline (Tris-buffered saline (TBS): 10 mM Tris-HCl, 150 mM NaCl, pH 7.5) containing 1% bovine serum albumin (BSA) for ≥2 h. The anti-GFP antibody (#G1112 Santa Cruz Biotechnology Inc. CA, USA) was incubated with the membranes at a 1∶200 dilution in TBS containing 3% BSA overnight at 4°C. After washing, a 1∶5000 dilution of anti-rabbit IgG HRP secondary antibody (#E1012 Santa Cruz Biotechnology) was incubated for 1 hour at room temperature in TBST containing 3% BSA. The filter was then washed and incubated with a WestDura Femto ECL kit (Fisher Scientific, Leicestershire, UK), and bands were visualized using a ChemiScope 3400 Mini Imaging System (Clinx Science Instruments Co., Ltd., China).

### Quantitative Real-time PCR and Semi-quantitative RT-PCR

Quantitative real-time PCR (qPCR) was used to analyze gene expression levels as described previously [41]. cDNA was synthesized using PrimeScript® RT Master Mix (TaKaRa, China) in a 20 µl-volume according to the manufacturer's instructions. The qPCR was carried out using primers JcLEA-F and JcLEA-R in SYBR® Premix Ex Taq™ II (Perfect Real-Time; TaKaRa, China) on a StepOnePlus™ Real-Time PCR System (Applied Biosystems) with three replicates. The PCR procedure was 95°C for 30 s, 40 cycles of 95°C for 5 s, and 60°C for 34 s, followed by 95°C for 15 s, 60°C for 1 min, and 95°C for 15 s. The *actin* gene from *J. curcas* was used as the internal control.

The relative expression of the *JcLEA* gene was analyzed in four transformed lines of Arabidopsis by semi-quantitative RT-PCR using the primers JcLEA-full-F and JcLEA-full-R with the following PCR protocol: 94°C for 5 min, followed by 28 cycles of 94°C for 30 s, and 55°C for 30 s. The *tubulin* gene from *Arabidopsis* was used as the internal control.

### Electrolyte Leakage Measurement

Ion leakage was determined by the method of Zhou et al. [42]. Leaves from four plants of each line were sampled for each treatment and rinsed with distilled deionized water to remove surface ions before being incubated in 8 ml deionized water at room temperature for 12 h. The sample conductivities (C1) were measured with a DDS-11A conductivity meter (Shanghai SUOSHEN Electrical Equipment Co. Ltd., China). The samples were then boiled for 1 h at 100°C and conductivities (C2) were measured when they had cooled to room temperature. The relative electrolyte leakage was calculated using the following formula: C1/C2 ×100%. The whole assay was repeated three times and the data analyzed using the Student's *t*-test.

### Relative Water Content Measurement

Leaves from four plants of each Arabidopsis line were collected for each treatment and the fresh weights (FW) were determined. The samples were then incubated in 8 ml deionized water at room temperature for 12 h at room temperature. The turgid weights (TW) of the leaf samples were measured. All samples were oven-dried at 65°C for 30 h, and the dry weights were measured. The relative water content (%) was calculated as = 100%×(FW-DW)/(TW-DW) [43]. Three replicates were performed, and the data was analyzed by Student's *t*-test.

### Glucose Content Assay

Glucose content was assayed with a Glucose (HK) Assay kit (Sigma-Aldrich, Inc.). Leaves from four different plants of each Arabidopsis line for each treatment were collected and incubated in 1.5 ml distilled deionized water and the absorbance of NADH at 340 nm versus distilled deionized water was measured using a BioPhotometer Plus (Eppendorf, Germany) [43]. Three replicates performed, and the data were analyzed by Student's *t*-test.

### Potassium and Sodium Ion Contents

A minimum of 30 plants from each transgenic line were treated with 50 mM, 100 mM, and 150 mM NaCl. Whole plants were washed three times with distilled deionized water to remove any possible surface ions before drying, and 0.5 g to 1 g tissues samples were then used for measurements. Samples were digested in 30 ml mixed acid digestion solution (HNO_3_:HClO_4_) and the mixed solutions were heated on the electro-thermal board to clarification. The potassium or sodium standard concentration liquid was diluted and added into flame photometer with the samples and the blank control. Potassium production was determined at 766 nm and sodium at 589 nm using a flame photometer (FP6410, China). The potassium and sodium contents of the samples were calculated against standard curves for potassium and sodium.

## Results

### Molecular Cloning of the Full-length cDNA of *JcLEA*

**Figure 1:**
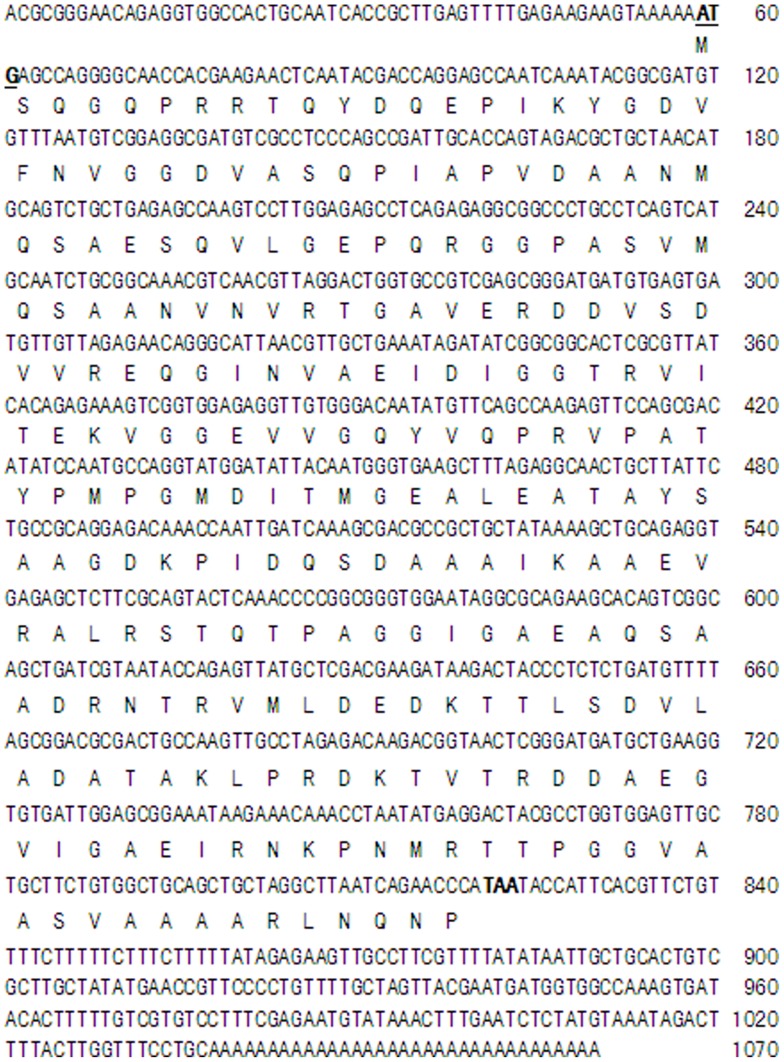
Nucleotide and deduced protein sequences of the cloned JcLEA gene. The start and stop codons are indicated in bold.

*J. curcas* belongs to the botanical family *Euphorbiaceae* and grows widely in tropical and sub-tropical areas of Central and South America, Africa, India, and Southeast Asia. *J. curcas* has been considered to be a type of crop that is highly tolerant to both dehydration [44] and high levels of salinity [37]. To understand the role of the LEA protein from *J. curcas*, we first cloned and sequenced the full-length *JcLEA* cDNA based on the highly conserved amino acid sequences of known plant LEA proteins in the NCBI database using the SMART™ RACE cDNA Amplification Kit (Clontech, Palo Alto, CA, USA). The full-length *JcLEA* cDNA comprised a 765 bp open reading frame (ORF), a 58 bp 5′-untranslated region (UTR), and a 247 bp 3′-UTR with a poly (A) tail (Fig. 1). The ORF encoded a polypeptide of 254 amino acids, for which the calculated isoelectric point and molecular mass were predicted to be 4.64 and 26.5 kDa, respectively (pI/Mw Tool at www.expasy.org). The predicted JcLEA protein contained 33.46% hydrophobic residues, including alanine, isoleucine, leucine, phenylalanine, and valine residues, and no cysteine, histidine or tryptophan residues.

### Homology Analysis of Amino Acid Sequences

**Figure 2:**
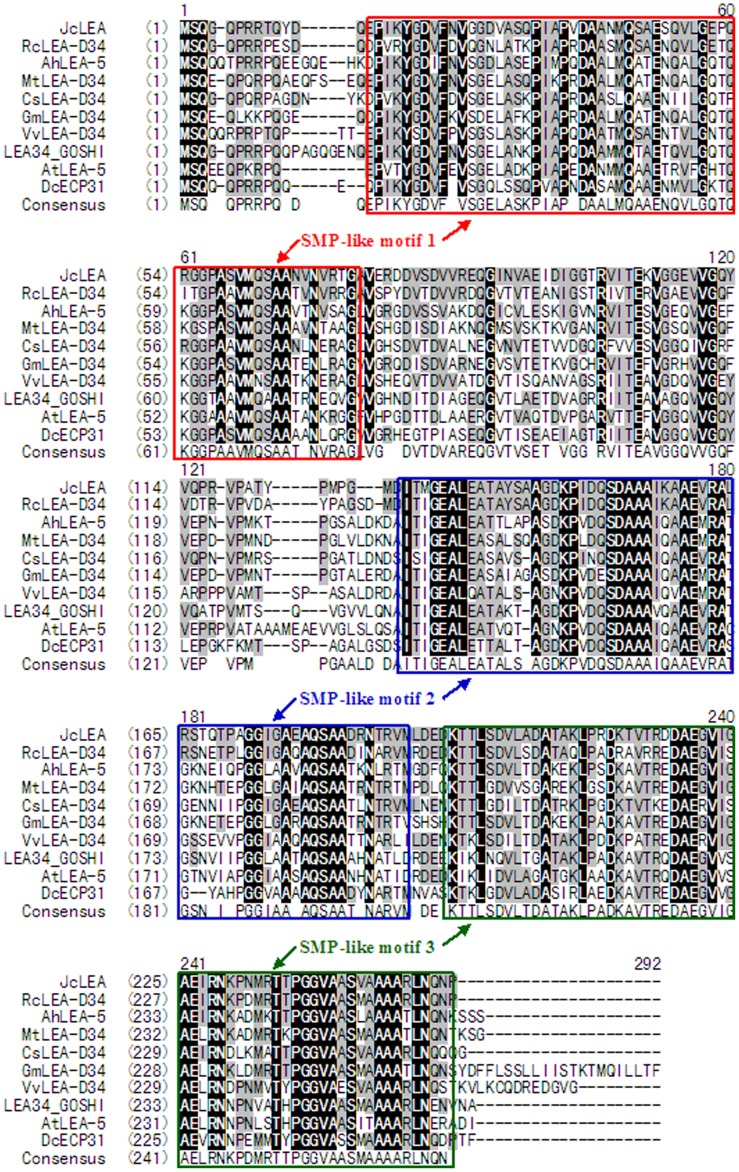
Alignment of JcLEA with other plant group 5 LEA proteins sequences. The alignment was performed using the published sequences of LEAs including RcLEA-D34 from *Ricinus communis* (XP_002520716), AhLEA-5 from *Arachis hypogaea* (ADQ91843), MtLEA-D34 from *Medicago truncatula* (XP_003590628), CsLEA-D34 from *Cucumis sativus* (XP_004146999), GmLEA-D34 from *Glycine max* (XP_003536910), VvLEA-D34 from *Vitis vinifera* (XP_003632304), LEA34_GOSHI from *Gossypium hirsutum* (p09444), AtLEA-5 from *Arabidopsis thaliana* (BAB01464), and DcECP31 from *Daucus carota* (BAD86645). Amino acid residues conserved in all sequences are boxed in black, while similar amino acids are boxed in gray. The three SMP-like motifs are indicated by red, blue, and green boxes.

A database search using Blastx (http://www.ncbi.nlm.nih.gov/) showed that there was a relatively high similarity between the predicted JcLEA protein and other plant LEA proteins. Notably, the LEAs identified in the blast analysis all belonged to the group 5 subfamily, and included the following nine plant proteins: RcLEA-D34 from *R. communis* (XP_002520716), AhLEA-5 from *Arachis hypogaea* (ADQ91843), MtLEA-D34 from *M. truncatula* (XP_003590628), CsLEA-D34 from *Cucumis sativus* (XP_004146999), GmLEA-D34 from *Glycine max* (XP_003536910), VvLEA-D34 from *Vitis vinifera* (XP_003632304), LEA34_GOSHI from *G. hirsutum* (p09444), AtLEA-5 from *A. thaliana* (BAB01464) and DcECP31 from *Daucus carota* (BAD86645). A number of gaps and insertions were needed to optimize the alignment. The amino acid identities between JcLEA and RcLEA-D34, AhLEA-5, MtLEA-D34, CsLEA-D34, GmLEA-D34, VvLEA-D34, LEA34_GOSHI, AtLEA-5, and DcECP31 were found to be 76%, 64%, 64%, 64%, 63%, 62%, 61%, 57%, and 57%, respectively. Moreover, three SMP-like motifs that are generally present in LEA proteins were also detected (Fig. 2).

### Phylogenic Analysis

**Figure 3:**
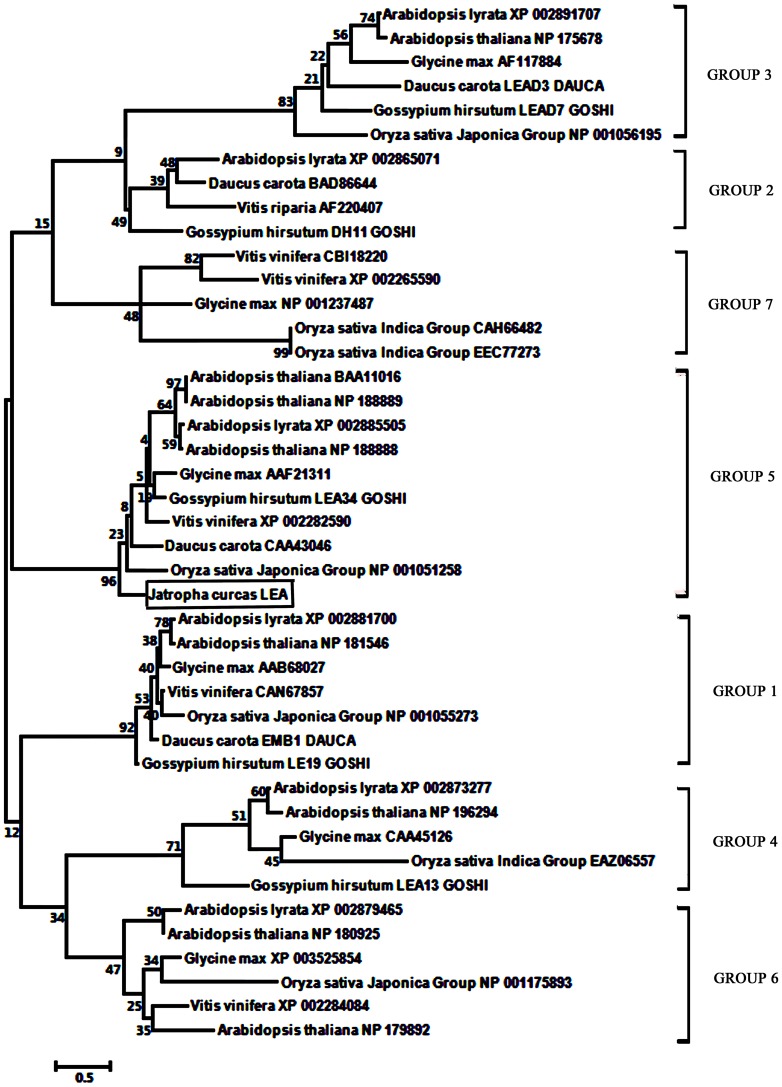
Phylogenetic tree of LEA protein sequences from different plant species. MEGA 5.0 was used to create an alignment of the protein sequences downloaded from the NCBI database (http://www.ncbi.nlm.nih.gov/). The JcLEA protein cloned from *J. curcas* shown boxed in group 5.

As there was a relatively high degree of similarity between JcLEA and the other group 5 LEA proteins, we constructed a phylogenetic tree to determine the specific group to which JcLEA belongs. The representative protein sequences of seven groups of LEA protein were used to perform Blast analyses so that LEA proteins from various plant species in each group could be identified. The maximum likelihood tree constructed with MEGA 5.0 showed that the JcLEA protein is related to group 5 LEA proteins and clustered together with the representative proteins of this group. In addition, we observed from the phylogenetic tree that the proteins within one group shared high levels of similarity, but that the similarities between groups were relatively low (Fig. 3).

### Expression Pattern of *JcLEA* under Different Abiotic Stress Treatments

**Figure 4:**
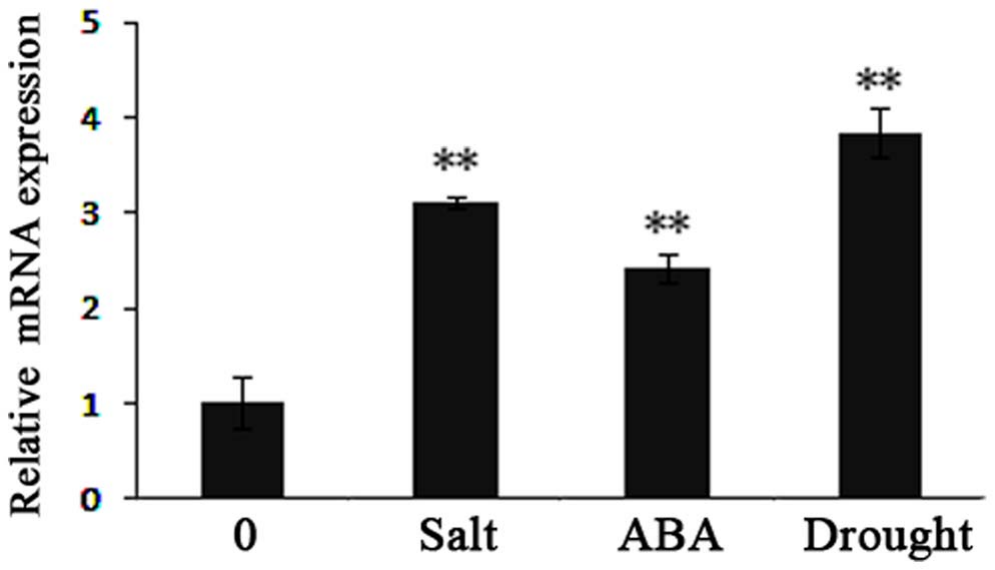
Quantitative real-time PCR analysis of *JcLEA* gene expression. Leaves of 30-day-old *J. curcas* plants were treated with 300 mM NaCl for one day, 100 µM ABA for three hours, and 30% PEG-4000 for three days, respectively.

Previous reports have shown that the expression of *LEA*-like genes could be induced by ABA, cold, drought, and high salt treatments [45], [46]. As *J. curcas* can tolerate dehydration and high levels of salinity, we have tested the possibility that *JcLEA* is involved in the plant response to drought and high salt. The gene expression patterns of *JcLEA* in response to 30% PEG for 3 days [47], 100 µM ABA for 3 hours [42], and 300 mM NaCl for 1 day [48] were investigated in young leaves of *J. curcas* seedlings. The relative expression of *JcLEA* was significantly increased in response to drought, ABA, and NaCl treatments, suggesting that *JcLEA* might play a role in dehydration and high salt tolerance (Fig. 4).

### Subcellular Localization of JcLEA Protein

**Figure 5:**
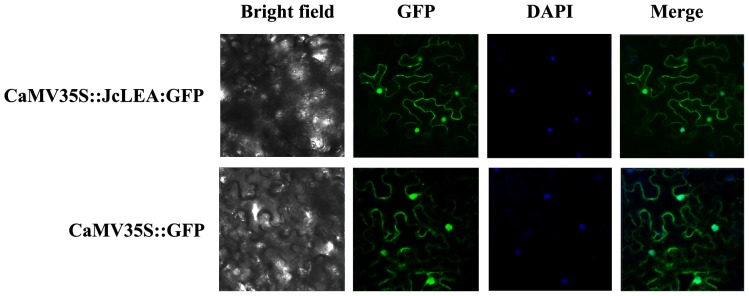
Subcellular localization of the JcLEA protein in tobacco leaf cells. GFP fluorescence of JcLEA: GFP and the GFP control are shown. DAPI staining was used to visualize the nucleus.

**Figure 6:**
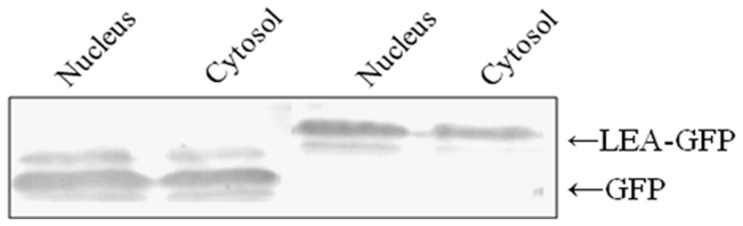
Detection of LEA-GFP fusions proteins extracted from tobacco leaves. Western blot analysis of proteins extracted from LEA-GFP or GFP transiently expressed in tobacco leaf cells, respectively.

Information on the subcellular localization of proteins can be important to elucidate the functional roles proteins play in plant cells [43]. In order to determine the localization of the JcLEA protein, the JcLEA-GFP translational fusion was constructed and transiently expressed under the control of the 35S promoter in tobacco leaves. Confocal laser fluorescence microscopy showed that the JcLEA-GFP fusion protein was localized to the cytosol and the nucleus (Fig. 5). In order to localize the JcLEA-GFP fusion protein more precisely, we tested 35S-JcLEA-GFP and 35S-GFP protein expression in transiently transformed tobacco, by extracting proteins and performing western blot analysis using a GFP polyclonal antibody (Fig. 6). These data showed that JcLEA-GFP was localized to both the cytosol and the nucleus, similar to the GFP control.

### Preparation of *JcLEA*-overexpressing *Arabidopsis* Lines

**Figure 7:**
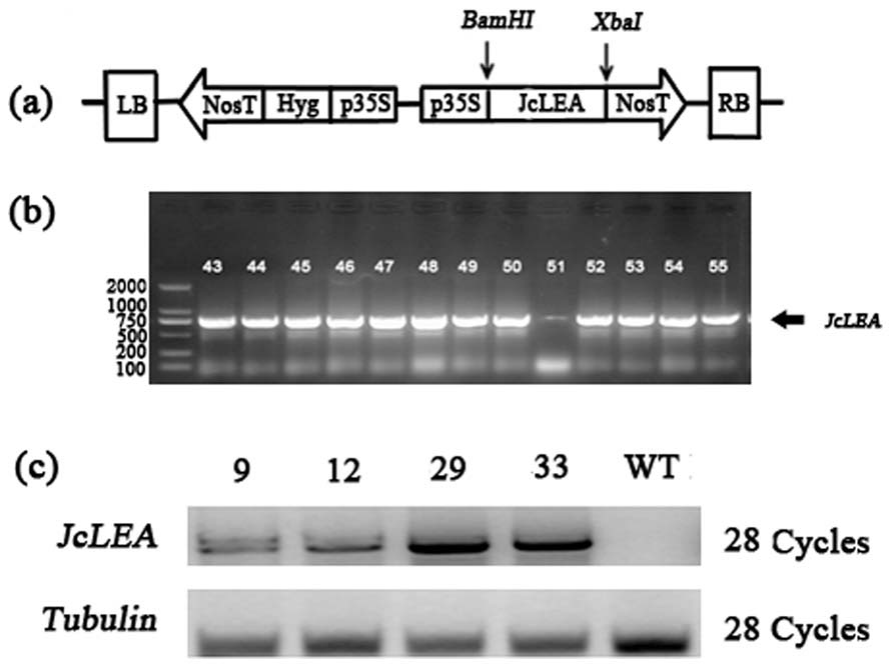
Incorporation and expression of *JcLEA* in transgenic Arabidopsis. (a) Schematic diagram of the *35S::JcLEA* DNA vector construct. (b) Verification of transformants from T_3_-generation regenerated *Arabidopsis* plants by PCR amplification from genomic DNA. Lane 1 contains a fragment size marker. (c) Expression levels of *JcLEA* in four transgenic lines as determined by semi-quantitative RT-PCR.

To study the contributions of *JcLEA* to drought and salt tolerance, we produced *JcLEA*-overexpressing lines of Arabidopsis Col-0 that were verified by genomic DNA PCR and semi-quantitative RT-PCR (Fig. 7a–c). The transgenic lines had different *JcLEA* expression levels. Among these, lines 25–29 and 25–33 showed higher expression levels of *JcLEA*, and *JcLEA* transcripts were not detected in the WT (Wild type) plants. Homozygous seeds of lines 25–29 and lines 25–33 were collected for further investigation. One set was germinated on soil for physiological measurements, and another set was germinated on MS medium for root length observation under conditions of drought and salt stress.

### Overexpression of *JcLEA* Enhanced Drought Tolerance in *Arabidopsis*

**Figure 8:**
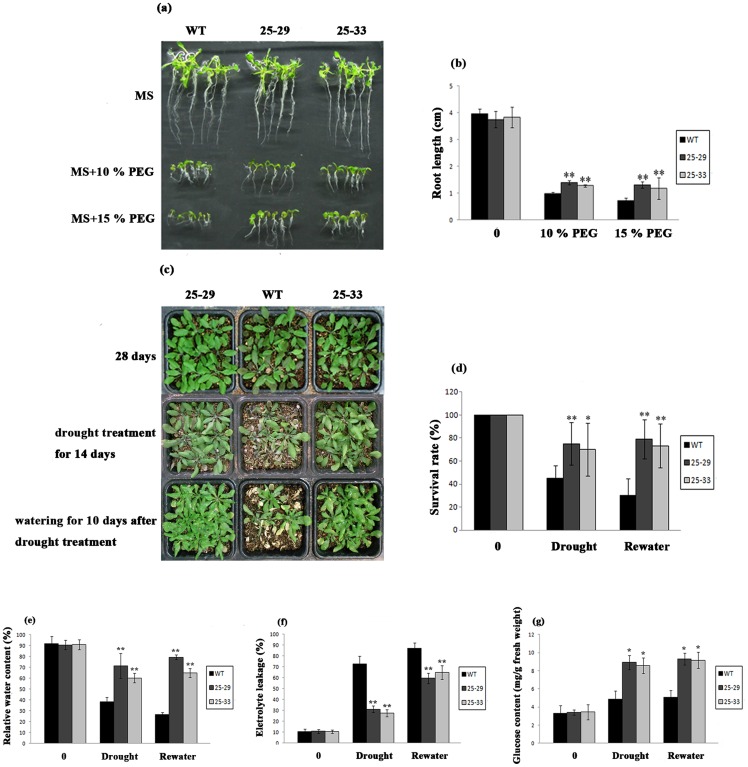
Phenotypes and physiological indices of transgenic *Arabidopsis* plants bioassayed for drought tolerance. (a and b) Phenotypes and root lengths of WT and transgenic plants treated with 10% and 15% PEG on MS medium (pH 5.8) at 22°C for two weeks. Error bars represent standard deviation (SD, n = 6). (c and d) Phenotypes and survival rates of plants after withholding water for 14 days, and plants that were then rewatered for 10 days. Representatives of typical plants are shown (SD, n = 3). (e and f) Measurement of relative water content, electrolyte leakage, and glucose content in plants on the 14^th^ day of dehydration treatment and the 10^th^ day after commencing rewatering (SD, n = 3). * *P*<0.05; ** *P*<0.01.

When the transgenic Arabidopsis seedlings were grown on MS medium containing 10% PEG and 15% PEG to be exposed to drought conditions, they exhibited much better growth status than did WT *Arabidopsis* plants, especially for root length, which is sensitive to dehydration (Fig. 8a & b). After water was withheld from the *Arabidopsis* plants for 14 days, the transgenic lines suffered less from dehydration compared with the WT plants, in which many of the leaves were bleached and withered (Fig. 8c). After watering again for 10 days, several of the withered leaves on the transgenic plants recovered and turned green, and the survival rates of the 35S::JcLEA seedlings were higher than those of WT (Fig. 8c & 8d). For further physiological investigation, the relative water content, electrolyte leakage, and glucose content were analyzed as markers of drought adaptation. The relative water content of transgenic *Arabidopsis* was significantly higher than that of WT drought treatment and drought plus rewatering, indicating that JcLEA could help to prevent water loss in plants (Fig. 8e). Electrolyte leakage, which is an indicator of the capacity to protect the plasma membrane integrity under stress, was also significantly lower in transgenic plants, demonstrating that JcLEA reduced damage to cell membranes in transgenic plants (Fig. 8f). Moreover, in response to dehydration stress, the transgenic plants accumulated more glucose, which contributed to the stability of the internal milieu of the plant cells (Fig. 8g). These physiological measurements indicated that the overexpression of *JcLEA* significantly improved the drought tolerance in *Arabidopsis*.

### Overexpression of *JcLEA* Enhanced Salt Tolerance in *Arabidopsis*

**Figure 9:**
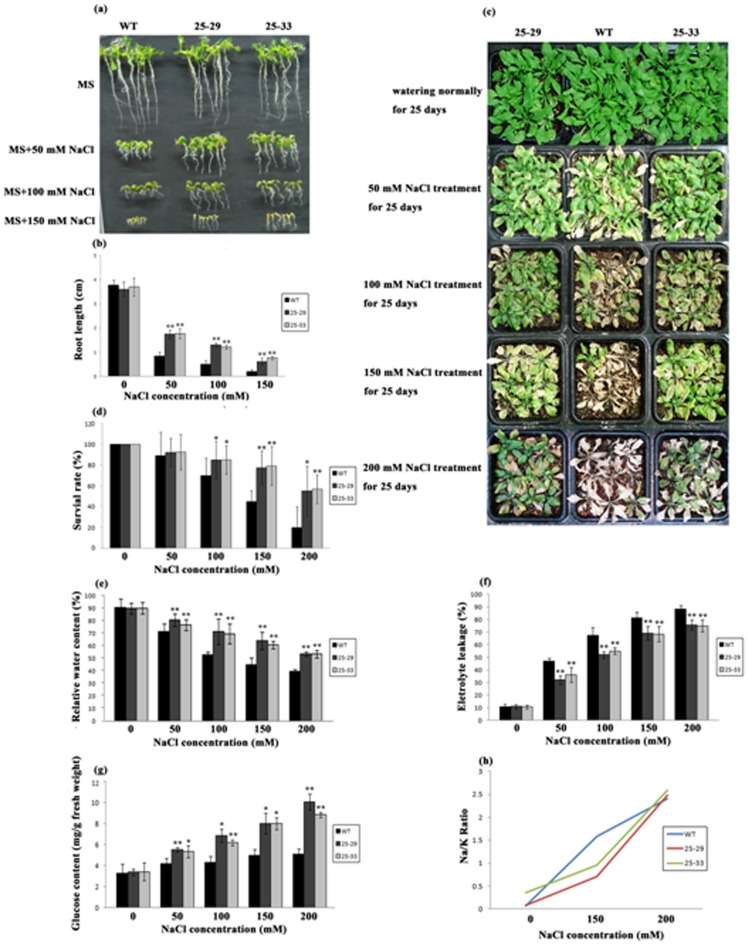
Phenotypes and physiological indices of transgenic *Arabidopsis* plants bioassayed for high salt tolerance. (a and b) Phenotypes and root lengths of WT and transgenic plants treated with 0, 50, 100, 150, and 200 mM NaCl on MS medium (pH 5.8) at 22°C for two weeks. Error bars represent the standard deviation (SD, n = 6). (c and d) Phenotypes and survival rates of plants treated with different concentrations of NaCl (SD, n = 3). (e and f) Measurements of relative water content (e), electrolyte leakage (f) and glucose content (g) in plants treated with different concentrations of NaCl (SD, n = 3). (h) The Na/K ratio of plants treated with 150 mM and 200 mM NaCl. * *P*<0.05; ** *P*<0.01.

Similar to the investigation of drought resistance, phenotypes and physiological indices in *35S::JcLEA* plants treated with NaCl were evaluated to test whether the JcLEA protein functions in salt tolerance. When the treated with NaCl solutions, the transgenic lines showed significantly higher levels of tolerance and higher survival rates than did the WT plants (Fig. 9a–d). WT plants displayed severe damage at ≥100 mM NaCl, while nearly 50% of the *35S::JcLEA* plants survived even when treated with 200 mM NaCl. The physiological indices including relative water content, electrolyte leakage, and glucose content also indicated that JcLEA contributed to cellular protection during salt stress (Fig. 9e–g). When exposed to high levels of NaCl, plant cells attempt to maintain high concentrations of K^+^ and low concentrations of Na^+^ in the cytosol in order to establish intracellular K^+^ and Na^+^ homeostasis, which is crucial for the protection of cytosolic enzymes and osmotic pressure stability [49]. We evaluated the Na/K ratio in transgenic and control seedlings based on the Na^+^ and K^+^ contents. Our results showed that presence of JcLEA reduced the Na/K ratio below 150 mM NaCl, suggesting a protective function for this protein through exclusion of extra Na^+^. However, JcLEA failed to exclude Na^+^ in the 200 mM NaCl treatment, which might be an upper limit for the capacity of ionic adjustment (Fig. 9h).

## Discussion

The LEA proteins are a family of hydrophilic proteins that are presumed to play a protective role during exposure to different abiotic stresses. This large protein family can be classified into seven groups, designated groups one to seven [22]. Among these, the roles of groups 1, 2, and 3 have been extensively studied in relation to the abiotic stress response. HVA1 from *Hordeum vulgare*, belonging to group 3, confers increased drought tolerance in wheat and rice [50], [51], and enhanced drought, salinity, and cold tolerance when expressed in mulberry [52]. In the group 2 LEAs, PMA80 from wheat increases dehydration tolerance of transgenic rice [53], and DHN24 from *Solanum sogarandinum* improves the cold tolerance of transgenic cucumber [54]. Similarly, the group 1 protein PMA1959 from wheat enhances tolerance to dehydration and salinity in transgenic rice [53]. On the other hand, some of the LEA proteins appear to contribute little to plant stress tolerance. The overexpression of two cold-induced LEA proteins CAP160 and CAP85 from *Spinacia oleracea* and three desiccation-induced pcC proteins from *C. plantagineum* failed to change the freezing or drought tolerance in transgenic tobacco plants [55], [56]. Thus, LEA family members may have different functions in plant stress responses. In comparison, the role of group 5 LEA proteins in abiotic stress tolerance in plants is not well known. Here, we show that a novel JcLEA protein isolated from *J. curcas* possesses high level of amino acid sequence identity and has similarity to other group 5 LEA proteins from plants. The ABA-, drought-, and salt-induced expression patterns of *JcLEA* are also typical of other *LEA*-like genes. Treatments with ABA, drought, salt, and extreme temperatures caused the accumulation of many type of LEA proteins [19], [34], [57], [58]. ABA is a key signaling phytohormone involved in the drought and salt responses in plants [8]. Previous reports have shown that the promoter regions of the group 5 LEA genes generally contain the ABA responsive element (ABRE), which causes the induction of these genes in response to various abiotic stress conditions [59]. The group 5 *JcLEA* genes are strongly induced by ABA, drought, and salinity, indicating a potential role for JcLEA in the response to drought and salt stress in *J. curcas*, which is consistent with the functions of characterized LEAs from other plant species [25].

In *Arabidopsis*, the protective function of JcLEA was confirmed, based on the plant growth status and physiological indices. JcLEA effectively prevents the loss of water and cytoactive components to maintain the integrity of plasma membranes and the homeostasis of plant cells under dehydration as well as high salt stress. The contribution of JcLEA to drought tolerance is consistent with the strong dehydration resistance observed in *J. curcas*. During salt stress, the accumulated excess Na^+^, which is toxic to enzymes, is extruded or compartmentalized in the vacuole to prevent growth cessation or cell death [60]. The measurements of potassium and sodium contents show that JcLEA may regulate Na^+^ transport and maintain the intracellular K^+^ concentration to stabilize the ionic balance of plant cells. The function of JcLEA is associated with its subcellular localization. It has been reported that LEA proteins, which are non-trans membrane proteins, are widely localized in multiple subcellular compartments in various plant species [19]. The PsLEAm protein from pea is localized in the mitochondrial matrix of seeds, and is the first mitochondrial presequence in a plant LEA protein [61]. Group 1 (AfrLEA1-1) and group 3 (AfrLEA3-4) LEA proteins from *Artemia franciscana* are also localized in mitochondria [62], while many kinds of group 2 LEA proteins are localized in the nucleus [22]. The respective subcellular locations of LEA proteins may be correlated with the different aspects of cellular protection during various abiotic stresses. JcLEA appears to be localized mainly to the nucleus and cytoplasm, which suggests that it has comprehensive protective functions in plant cells. This finding is also consistent with the water and ionic homeostasis in transgenic Arabidopsis plants that express JcLEA.

In summary, JcLEA is a novel LEA protein belonging to group 5. The constitutive expression of *JcLEA* does not cause growth repression in host plants. From the results of this study, it can be concluded that JcLEA has a huge potential for transgenic breeding based on (1) the fact that the gene is induced by exposure to drought and salt, (2) the subcellular localization of JcLEA and (3) the improved tolerance to dehydration and high salt levels observed in transgenic *Arabidopsis* plants.
